# The diversity of cytomegalovirus among blood donors and transplant recipients could affect the effectiveness of specific anti-CMV immunoglobulins

**DOI:** 10.3389/fimmu.2026.1832716

**Published:** 2026-04-29

**Authors:** Stefania Braidotti, Debora Curci, Chiara Rodaro, Anna Flamigni, Alessandra Maestro, Davide Zanon, Antonello Di Paolo, Natalia Maximova

**Affiliations:** 1 Department of Pediatrics, Institute for Maternal and Child Health - IRCCS “Burlo Garofolo” – Trieste, Italy; 2 Advanced Translational Diagnostic Laboratory, Institute for Maternal and Child Health - IRCCS “Burlo Garofolo” – Trieste, Italy; 3 Clinical Department of Medical, Surgical and Health Science, University of Trieste, Trieste, Italy; 4 Pharmacy and Clinical Pharmacology Department, Institute for Maternal and Child Health - IRCCS “Burlo Garofolo” – Trieste, Italy; 5 Section of Pharmacology, Department of Clinical and Experimental Medicine, University of Pisa, Pisa, Italy

**Keywords:** CMV prophylaxis, CMV-specific immunoglobulin, hematopoietic stem cell transplantation, pediatric, population pharmacokinetics

## Abstract

**Introduction:**

Human cytomegalovirus (CMV) remains a major cause of morbidity and mortality in pediatric allogeneic hematopoietic stem cell transplantation (allo-HSCT), where its high genetic variability and the limitations of current antiviral therapies pose significant clinical challenges. Despite standard antiviral prophylaxis, CMV infection affects up to 80% of allo-HSCT recipients, necessitating the evaluation of alternative strategies to overcome the limitations of conventional treatments. This single-center ambispective study investigates the application of CMV-specific immunoglobulin (Ig) prophylaxis, focusing on its impact on viral clearance, transplant outcomes, immunological readouts, and efficacy across diverse ethnic cohorts (European vs. non-European).

**Methods:**

A *post hoc* analysis was performed on 71 hematopoietic stem cell recipients who received CMV-specific Ig (Study Group) and historical controls (Control Group). A pharmacokinetic analysis was performed on the Study Group.

**Results:**

The Control Group exhibited higher rates of first-blood CMV DNA detection post-transplant (p = 0.0024), second-blood CMV DNA detection post-transplant (p = 0.00042), CMV DNA viral load (p = 0.0016), reduced hospital stays (p = 0.007) and improved immune reconstitution (p < 0.0001). No significant differences in hospital stay emerged between European and non-European patients. However, Europeans had better results in first-blood CMV DNA detection (p = 0.006), second reactivation (p = 0.00032), immune reconstitution at day +90 (p = 0.0443), clearance rates, and reduced need for second-line therapy (p < 0.001). The terminal half-life of CMV-specific Ig was approximately 15 days.

**Discussion:**

These findings demonstrate that CMV-specific Ig serves as an effective molecular immunotherapy, significantly improving immunological readouts and clinical outcomes in pediatric allo-HSCT. Furthermore, the observed ethnic disparities underscore the importance of personalized molecular strategies to address CMV genome variability and global health challenges.

## 1 Introduction

Human cytomegalovirus (CMV) is a geographically ubiquitous human-restricted herpesvirus. Depending on the geographical region and increasing with age, CMV establishes a lifelong latent infection following a primarily asymptomatic primary infection in 45–100% of adults (1). Reactivation of the latent virus is generally not associated with disease in immunocompetent individuals. Conversely, in immunocompromised transplant recipients, CMV reduces graft survival and increases the risk of multiorgan disease, graft-versus-host disease (GVHD), infection with other pathogens, post-transplant lymphoproliferative disorders, and transplant-related mortality (TRM) (2). Despite the administration of antiviral prophylaxis, the incidence of CMV infection following allogeneic hematopoietic stem cell transplantation (allo-HSCT) remains at 37.5–80% (3–5). In addition to a lack of sufficient efficacy, anti-CMV drugs are associated with severe side effects, including bone marrow suppression and renal impairment (6). The low efficacy of CMV-specific treatment is partly due to the substantial genetic variability observed across CMV genomes, which is higher than that observed in any other human herpesvirus studied (7). This elevated nucleotide diversity is attributed to high intrahost rates of *de novo* mutation (8). In addition to high-strain complexity, the diversity of CMV is attributed to the high frequency of multi-strain CMV infections involving genetically distinct strains (9).

The role of virus-specific antibodies in protecting against CMV infection remains a topic of discussion. Recent studies indicate that neutralizing antibody titers correlate with protection from infection in transplant recipients (10). In contrast, other studies have shown that only the T-cell response is associated with protection from CMV infection in transplant recipients (11, 12). A study conducted on an animal model demonstrated that humoral immunity alone can protect against viral reactivation in the post-transplant period (13). Despite the absence of T and NK cells, mouse CMV was not detected in latently infected mice that were transplanted with T-cell-replete grafts. These mice were further protected from viral reactivation with the adoptive transfer of immune serum. This evidence is further supported by observations in solid-organ transplant recipients, wherein elevated epithelial cell-neutralizing antibody titers (i.e., >480) have been correlated with a reduced incidence of CMV infection, a decreased duration of treatment, and complete protection from CMV disease (10). Moreover, the employment of a CMV strain-specific immune serum demonstrated efficacy in a pediatric HSCT recipient with an unfavorable prognosis for CMV disease. The failure to respond to conventional antiviral treatments was due to the concurrent presence of at least four native South Asian CMV strains (14).

The use of anti CMV-specific immunoglobulin (Ig) is consequently controversial in post-transplant clinical practice. Our Transplant Centre has a decade-long experience with the use of specific anti CMV-specific Ig for post-transplant prophylaxis and as a second-line treatment. The primary aim of our study was to evaluate the impact of CMV-specific Ig prophylaxis on the incidence and severity of CMV infection in pediatric allo-HSCT recipients. Secondary objectives included the assessment of the potential impact of CMV-specific Ig prophylaxis on transplant-related outcomes and the determination of its comparable efficacy across diverse ethnic groups.

Furthermore, a population pharmacokinetic (POP/PK) analysis was performed to describe the disposition and elimination of anti-CMV Ig in patients and to identify any potential factors affecting pharmacokinetics.

## 2 Materials and methods

### 2.1 Study design and population

This single-center ambispective study enrolled pediatric allo-HSCT patients at the Pediatric Bone Marrow Transplant Center, Institute for Maternal and Child Health - IRCCS “Burlo Garofolo” – Trieste, Italy. The study was approved by the Institutional Ethics Committee (ref. 1105/2015; ClinicalTrials.gov NCT07013370) and written parental consent was obtained at admission for all participants.

The primary aim of the study was to assess the impact of CMV-specific Ig prophylaxis on clinical outcomes. The study population was comprised of two distinct groups, designated as the Study Group and the Control Group.

The Study Group included 71 pediatric patients aged 30 days to 18 years who underwent allo-HSCT from any donor source for malignant or non-malignant hematological diseases between June 2016 and December 2023. All Study Group patients received CMV-specific Ig prophylaxis as part of their standard transplant protocol during the enrolment period.

A *post hoc* comparison was performed using a historical Control Group consisting of 70 pediatric allo-HSCT recipients treated between January 2010 and May 2016 under identical institutional standard practices. The Control Group patients satisfied all the Study Group’s inclusion criteria and would have been eligible for CMV-specific Ig prophylaxis had it been available during their treatment period. This historical comparison enabled us to evaluate the clinical impact of the prophylactic strategy by comparing outcomes in patients treated before the introduction of CMV-specific Ig prophylaxis with those receiving it. Patients were eligible for inclusion if they underwent their first allo-HSCT for hematological disease under standard institutional transplant protocols and had complete follow-up data available. Exclusion criteria included: severe Ig-related adverse reactions, CMV reactivation prior to the initiation of CMV-specific Ig prophylaxis, and receipt of adoptive cellular post-HSCT immunotherapy during the study period. The exclusion criteria were applied with the objective of ensuring the homogeneity of the study population, thus avoiding the confounding factors associated with Ig tolerance and competing CMV management strategies.

Both groups exhibited comparable demographics, including age, gender, ethnicity, diagnosis, conditioning, donor type, stem cell source, and CMV serostatus.

Furthermore, the Study Group was divided into European (EU Group) and non-European subgroups (Non-EU Group). This classification refers specifically to the patients’ region of origin (the European continent) and is used to investigate potential regional differences in outcomes within the Study Group.

A schematic representation of the methods is provided in the **Supplementary Materials**, **Supplementary Figure 1**.

### 2.2 Transplant protocols and supportive care

All patients received standard myeloablative conditioning with chemotherapy and radiation (15). GVHD prophylaxis was performed with tacrolimus (16). Additional GVHD prophylaxis included rabbit antithymocyte globulin (ATG) and mycophenolate mofetil for the matched unrelated donor (MUD). Unmanipulated *in vivo* T-cell depletion with post-transplant cyclophosphamide (PTCy) platform of haploidentical HSCT has recently been replaced by an ex vivo TCR αβ+/CD19+ cell depletion plus memory T-cell add-back. All patients who received ex vivo GVHD-prevention manipulation strategies and memory T-cell add-back were strictly excluded from the Study Group analysis.

Prevention and treatment of infection, along with other elements of transplant-specific supportive care, were performed in accordance with institutional standard practices that have undergone minimal modifications since 2010. Notably, the protocols for antimicrobial, antifungal, and antiviral prophylaxis remained unchanged. In particular, the antiviral prophylaxis administered to subjects in both the Control and Study Groups comprised high-dose acyclovir, guided by therapeutic drug monitoring (TDM) (17).

The IgG replacement scheme has remained unchanged for the past two decades. Patients admitted to the Transplant Center who have been diagnosed with hypogammaglobulinemia received IgG replacement therapy prior to undergoing conditioning. Subsequently, transplant recipients received an IgG dose of 400 mg/kg on a 10-day schedule.

Furthermore, the blood transfusion policy, GVHD prophylaxis, donor selection, and the necessity for an aseptic environment remained unaltered throughout the years.

The modifications to the HSCT procedure from the Control Group to the Study Group included the following: the addition of CMV-specific IgG to the standard supplementation with non-specific IgG, the use of ruxolitinib for GVHD treatment, and innovative approaches for haploidentical transplant, such as an αβ+ T cell-depleted graft, post-transplant T memory cell addition for rapid immune reconstitution, or NK boost for uncontrolled viral and fungal infection. However, patients who received adoptive cell therapy were excluded from Study Group.

### 2.3 Clinical outcome definitions

Acute and chronic GVHD were diagnosed and graded using standard criteria by (18–20). The incidence of GVHD was defined as any GVHD requiring systemic immunosuppressive therapy. Early transplant-related complications were defined as events occurring within 100 days after HSCT that were not related to primary disease recurrence. The duration of follow-up was defined as the time interval from HSCT to the last contact or death. The minimum follow-up period for surviving patients was 12 months. One of the outcomes of interest was immune reconstitution, defined as the repopulation of CD4+ T-lymphocytes. A CD4+ T-lymphocyte count of at least 500 cells/μL in two consecutive measurements within 100 days after HSCT was considered successful immune reconstitution. Patients who died before 100 days of follow-up were assessed until the date of death.

### 2.4 CMV definitions and surveillance strategy

The diagnosis of CMV disease followed the criteria of the Transplant-Associated Virus Infections Forum. Post-transplant CMV-related complications were defined according to Ljungman et al. (21). CMV infection was defined as the isolation or detection of viral antigens or nucleic acids in any specimen. CMV DNAemia refers explicitly to the presence of any detectable amount of CMV DNA in plasma, serum, or whole blood, as measured by real-time polymerase chain reaction (PCR)-based techniques. The treatment started either when the DNA level was greater than 200 copies/mL at the first measurement, or when the viral load remained constant (<200 copies/mL) or increased (e.g., a twofold increase or an increase of ≥ 0.5 log_10_ copies/mL) at the second evaluation performed within 72–96 hours.

First-blood CMV DNA detection was defined as the initial detection of the virus in patients without prior exposure; second-blood CMV DNA detection was defined as a new episode that occurred after ≥4 weeks with no virus detection during surveillance. Refractory infection was defined as an absence of response, as indicated by a >1 log10 increase or <1 log10 decrease in viral load despite ≥2 weeks of antiviral therapy.

Post-transplant CMV DNAemia was measured in whole blood by real-time PCR using the CMV ELITe MGB Kit (ELITechGroup, Torino, Italy). Monitoring occurred twice weekly during conditioning to discharge, then weekly or at outpatient visits until immune reconstitution and discontinuation of immunosuppression.

### 2.5 CMV-specific Ig prophylaxis protocol and anti-CMV-specific treatment

CMV-specific Ig prophylaxis was administered exclusively to patients in the Study Group with commercially available preparation (Cytomegatect^®^, Biotest Pharma GmbH, Dreieich, Germany), produced in compliance with European Union safety and quality standards (Directive 2002/98/EC). Prophylaxis was administered twice a week, initiated on day 3 of conditioning and continued until discharge. Thereafter, CMV-specific Ig was administered at each admission until CD4+ normalization. If blood CMV DNA appeared during prophylaxis, administration was carried out for 3–5 consecutive days (boost) at the physician’s discretion, based on individual risk factors. Patients with no viral load decline or increased titers after boost were considered non-responders, and prophylaxis was discontinued. In CMV disease, dosage and duration were individualized according to clinical status and infection severity. CMV-specific Ig was infused over one hour at weight-based doses: 1000 U (<10 kg), 2000 U (10–25 kg), 3000 U (25–50 kg), and 4000 U (>50 kg).

Foscarnet was used as a first-line anti-CMV therapy during the first month following transplantation; ganciclovir or valganciclovir were administered post-engraftment. Second-line treatments included ganciclovir (replacing foscarnet) or vice versa, cidofovir, letermovir, and maribavir. Advanced-line therapy employed NK and CD45RO+ lymphocytes as adoptive cell therapy infusion. Total CMV IgG was quantified 24–72h after each inpatient or before outpatient infusion.

### 2.6 Population pharmacokinetic analysis

Plasma anti-CMV Ig concentrations were analyzed using a nonlinear mixed-effect method with Monolix software, version 2023.1 (Lixoft, Anthony, France), following standard procedures. Mono-, bi-, and tri-compartment models with different error structures were tested. Variabilities between- and within-subject as well as covariates (age, body weight, creatinine clearance), were evaluated for model inclusion. Improvements in PK parameter estimates guided model development, reductions in objective function value (OFV; forward inclusion >3.81, backward exclusion >6.63), and lower ETAs and relative standard errors. Graphical diagnostics (goodness-of-fit plots, residual distribution, pcVPC) supported selection. Individual half-life (t1/2,i) was calculated as (0.693×Vi)/Cli.

### 2.7 Statistical analysis

Continuous variables were summarized as the mean for normally distributed data and the median with interquartile range (IQR) for non-normal data. Qualitative variables were expressed as frequencies and percentages. Demographic and clinical characteristics were compared using the chi-square (χ²) test. Normality was assessed with the Shapiro–Wilk test. Univariate analysis of continuous variables used the parametric Wilcoxon test. Time-to-event analysis for clearance achievement was performed using the Kaplan–Meier curves, and differences between groups were assessed with the log-rank test. Differences in PK parameters across occasions were evaluated using analysis of variance (ANOVA). A p-value < 0.05 was considered statistically significant.

## 3 Results

### 3.1 Patient characteristics

Patient characteristics are summarized in **Table 1**.

**Table 1:** Patient characteristics.

Variables	CMV-specific Ig Group (n = 71)	Control Group (n = 70)	*p*-value
Age at transplant, years, median (IQR)	9.89 (5.48 – 13.83)	8.11 (4.76 – 11.52)	0.132
Sex, number (%):			
- male	45 (63.4)	47 (67.1)	0.948
Primary diagnosis, number (%):			
- acute leukemia	46 (64.8)	49 (70.0)	0.299
- myelodysplastic syndrome	10 (14.1)	8 (11.4)	
- solid tumor	3 (4.2)	2 (2.8)	
- non-malignant disease	12 (16.9)	11 (15.7)	
Patient’s origin, number (%):			
- European	32 (45.1)	34 (48.6)	0.065
- non-European	39 (54.9)	36 (51.4)	
Donor/Recipient CMV serostatus, number (%):			
- positive/positive	35 (49.3)	36 (51.4)	0.072
- negative/positive	14 (19.7)	13 (18.6)	
- positive/negative	8 (11.3)	6 (8.6)	
- negative/negative	14 (19.7)	15 (21.4)	
Donor type, number (%):			
- sibling	28 (39.4)	30 (42.8)	0.120
- MUD	33 (46.5)	34 (48.6)	
- haploidentical	10 (14.1)	6 (8.6)	
Type of conditioning, number (%):			
- TBI-based	36 (50.7)	39 (55.7)	0.06
- Busulfan/treosulfan-based	35 (49.3)	31 (44.3)	
Graft source, number (%):			
- bone marrow	18 (25.4)	27 (38.6)	0.131
- peripheral stem cells	53 (74.6)	43 (61.4)	
GVHD prophylaxis, number (%):			
- tacrolimus	28 (39.4)	30 (42.8)	0.08
- tacrolimus + MMF	33 (46.5)	34 (48.6)	
- tacrolimus + MMF + PTCy	5 (7.0)	6 (8.6)	
- none	5 (7.0)	0	
ATG used, number (%)	37 (52.1)	39 (55.7)	0.47

ATG, antithymocyte globulin; CMV, human cytomegalovirus; Ig, immunoglobulin; IQR, interquartile range; MUD, matched unrelated donor; TBI, total body irradiation; GVHD, graft versus host disease; MMF, mycophenolate mofetil; PTCy, post-transplant cyclophosphamide.

### 3.2 CMV reactivation and viral load

**Figure 1:**
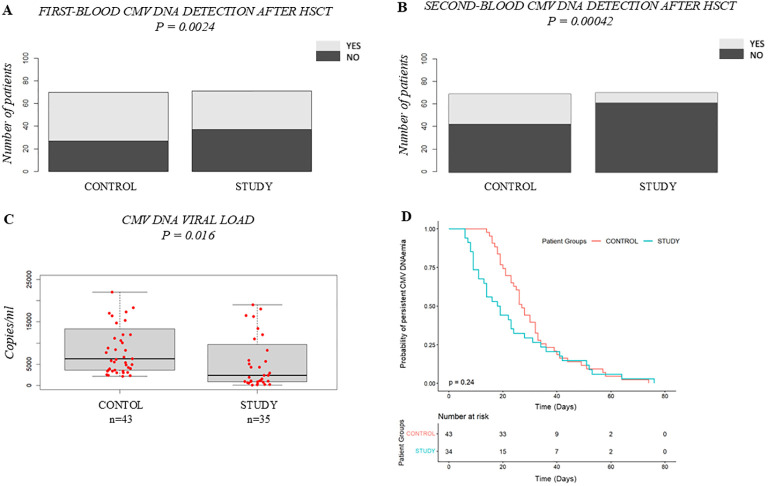
Efficacy of CMV-specific Ig prophylaxis: Control Group vs. Study Group. **(A)** Comparison of the incidence of patients with first-blood CMV DNA detection after HSCT; **(B)** Comparison of the incidence of second-blood CMV DNA detection after HSCT; **(C)** Boxplot comparing CMV DNA viral load (copies/mL); **(D)** Kaplan–Meier curves comparing the time to CMV DNAemia clearance. In **(C)** the bold horizontal line represents the median value; Control Group n=43, Study Group n=35. P-values were obtained using the χ2 test for categorical variables **(A, B)**, the Wilcoxon test for continuous variables **(C)** and the log-rank test for time-to-event analysis **(D)**. CMV, human cytomegalovirus; HSCT, hematopoietic stem cell transplantation.

CMV-specific Ig prophylaxis reduced CMV blood detection in pediatric allo-HSCT recipients. First-blood CMV DNA detection was higher in the Control Group (61.4%) than in the Study Group (47.8%, p = 0.0024) (**Figure 1A**). Second-blood CMV DNA detection occurred in 27 Control patients versus 9 Study patients (p = 0.00042) (**Figure 1B**). Median CMV DNA viral load were lower in the Study Group (2400 copies/mL, IQR 8780) than controls (6300 copies/mL, IQR 9750, p = 0.0016) (**Figure 1C**), with comparable viral clearance (p = 0.24) (**Figure 1D**). No differences were observed in time to CMV DNAemia detection from HSCT (p = 0.09) or second-line therapy use (p = 0.8729) (**Supplementary Figures 2A, B**).

### 3.3 Length of hospital stay and immunological recovery

**Figure 2:**
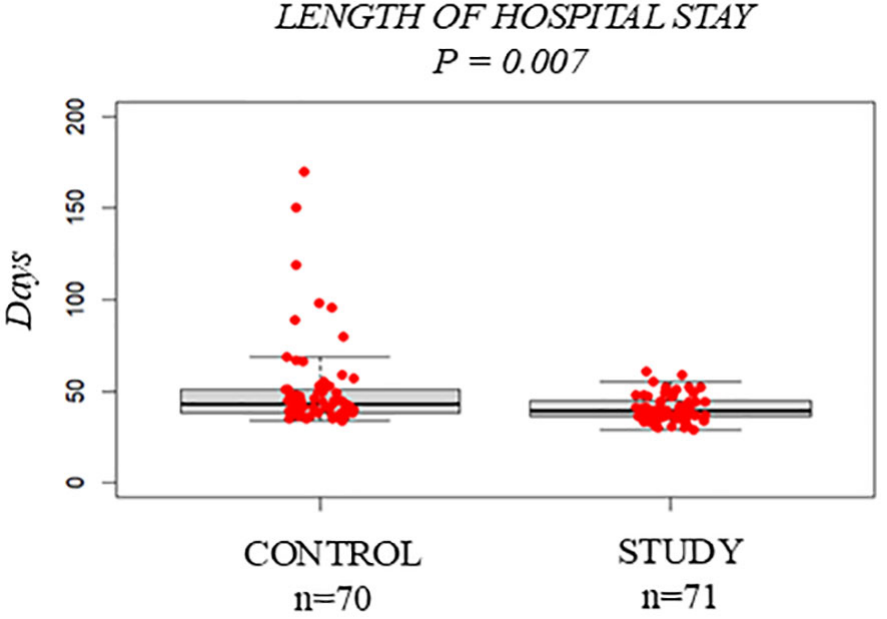
Transplant-related outcomes: Control Group vs. Study Group. Boxplot comparing the number of hospitalization days required for the Control Group and the Study Group. The bold horizontal line represents the median value; Control Group n=70, Study Group n=71. P-values were obtained using the Wilcoxon test.

**Figure 3:**
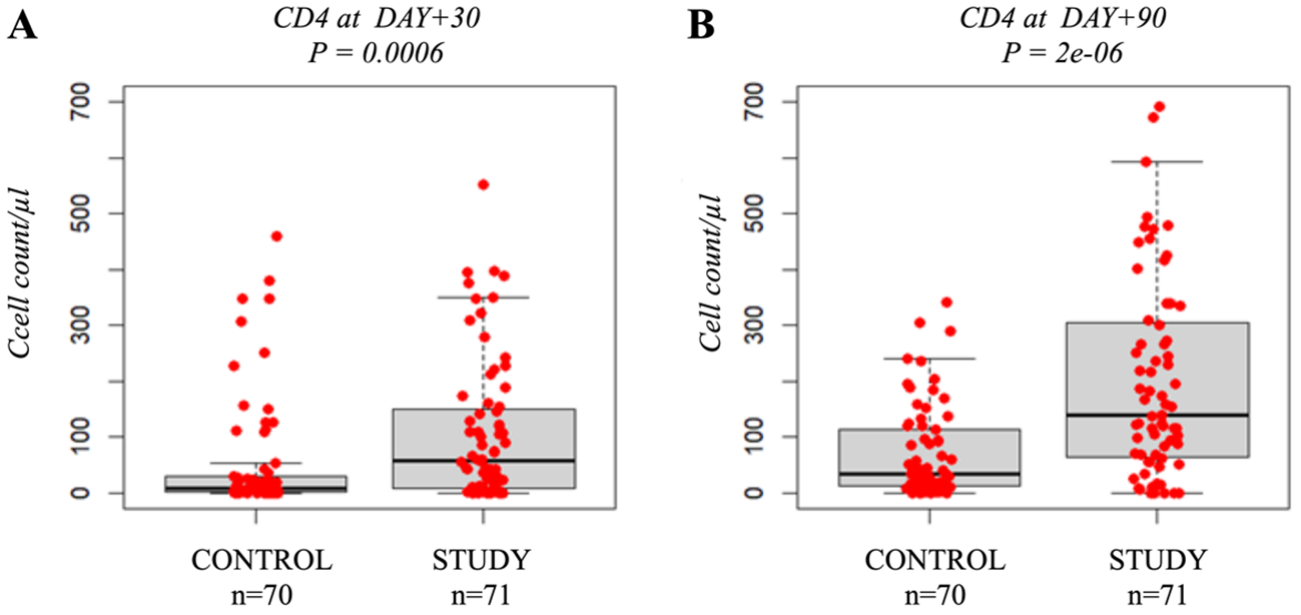
Immunological recovery after allo-HSCT: Control Group vs. Study Group. Boxplot comparing the median CD4+ count (cells/µL) at day+30 **(A)** and day+90 **(B)**. The bold horizontal line represents the median value; Control Group n=70, Study Group n=71. P-values were obtained using the Wilcoxon test.

Hospital stay was shorter in the Study Group (median 39 days, IQR 9.0) than in controls (43 days, IQR 12.8, p = 0.007) (**Figure 2**). Patients in the Study Group restored T-lymphocytes significantly faster than controls. At day +30, median CD4+ counts were 58 cells/µL (IQR 143.0) versus 9.5 cells/µL (IQR 27.3, p = 0.0006) (**Figure 3A**), increasing further at day +90 (139 vs. 35.5 CD4+/µL, p = 2e-06) (**Figure 3B**). No significant differences were observed between groups in acute or chronic GVHD (p = 0.2937 and p = 0.5925) (**Supplementary Figures 3**, **4**).

### 3.4 Impact of ethnicity on immune reconstitution

**Figure 4:**
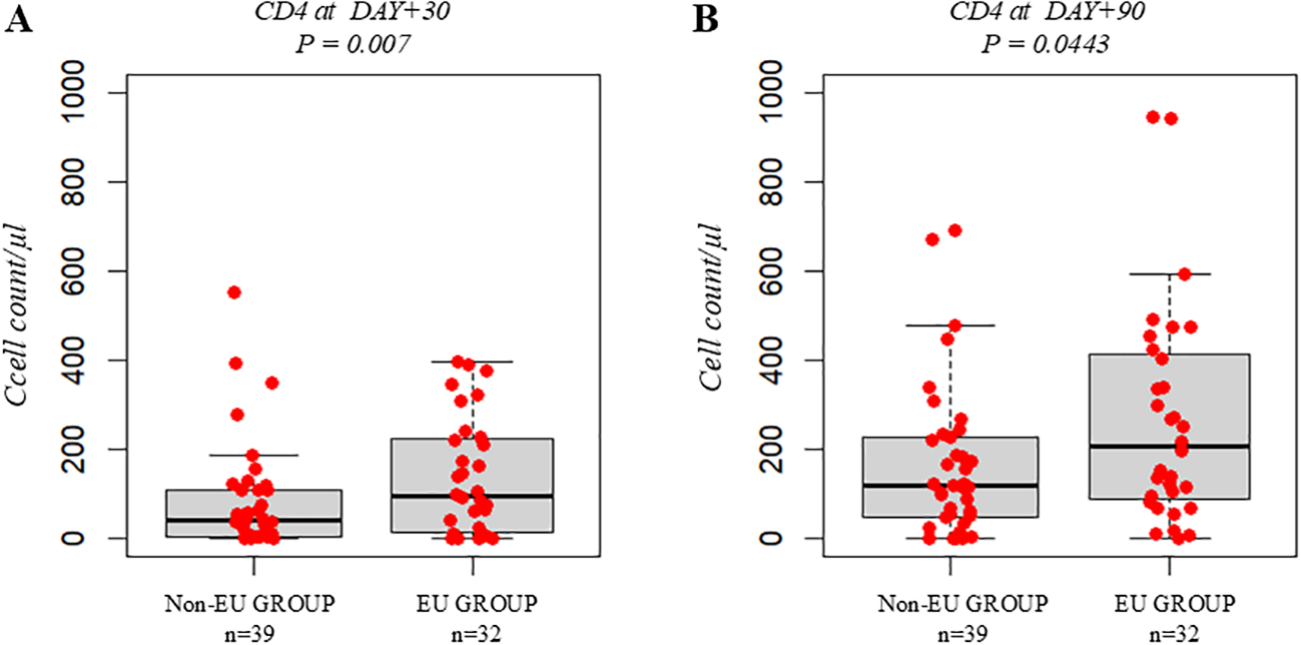
Immunological recovery after allo-HSCT: Non-European Group vs. EU Group (belonging to Study Group). Boxplot comparing the median CD4+ count (cells/µL) at day+30 **(A)** and day+90 **(B)**. The bold horizontal line represents the median value; Non-European Group n=39, EU Group n=32. P-values were obtained using the Wilcoxon test.

Within the Study Group, patients from the EU (n = 32) and non-European countries (n = 39) showed similar hospital stay (p = 0.8 and p = 0.1774), as well as comparable day +30 immune recovery (p = 0.0768). However, at day +90, EU patients exhibited higher CD4+ counts (median 206 vs. 118.5 cells/µL, p = 0.0443) (**Figures 4A, B**).

### 3.5 Ethnicity-dependent differences in CMV reactivation and viral load

**Figure 5:**
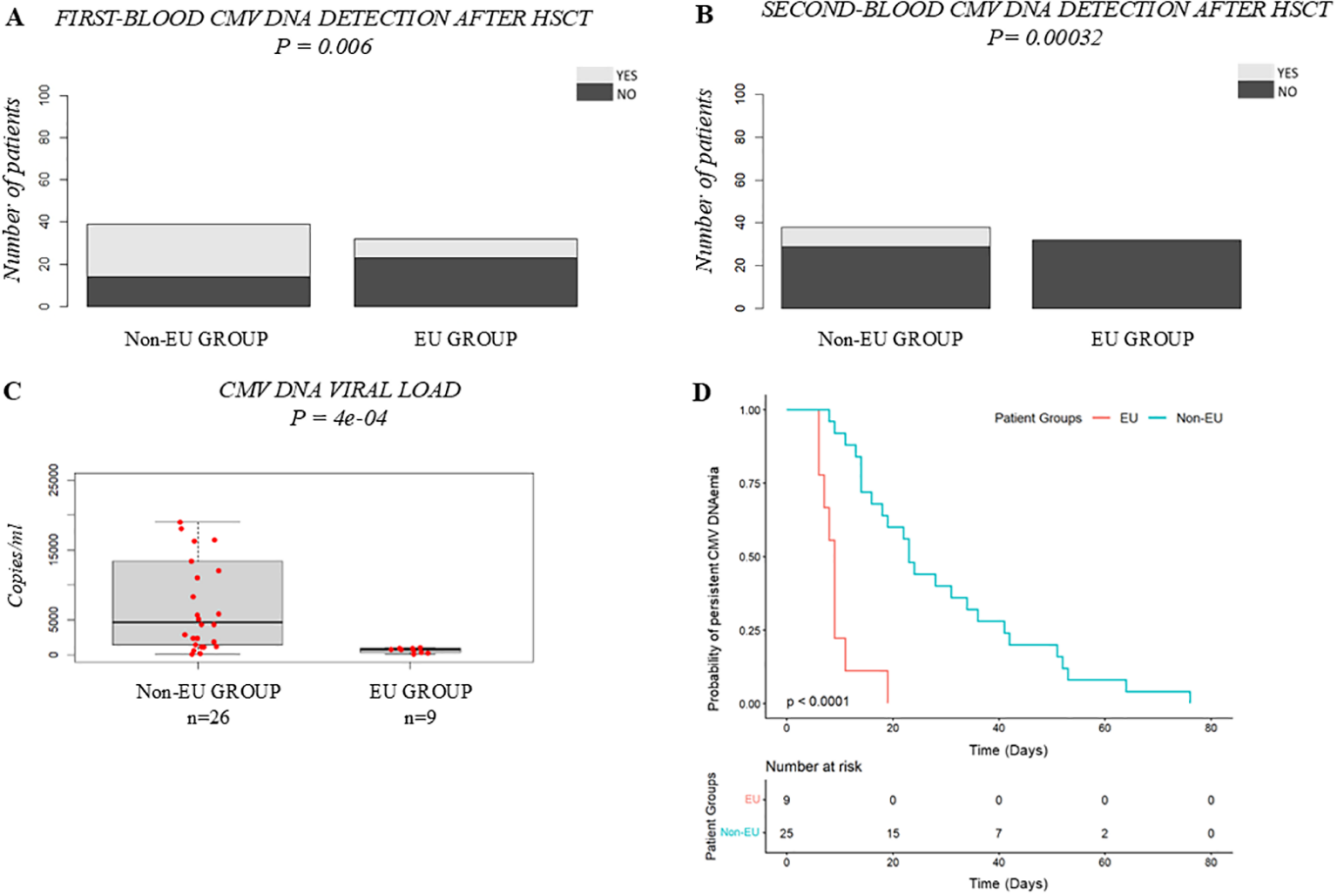
Efficacy of CMV-specific Ig prophylaxis: Non-European Group vs. European Group (belonging to Study Group). **(A)** Comparison of the incidence of patients with first-blood CMV DNA detection after allo-HSCT; **(B)** Comparison of the incidence of second-blood CMV DNA detection after allo-HSCT; **(C)** Boxplot comparing CMV DNA viral load (copies/mL); D) Kaplan–Meier curves comparing the time to CMV DNAemia clearance. In Panel C the bold horizontal line represents the median value; Extra-EU Group n=26, EU Group n=9. P-values were obtained using the χ2 test for categorical variables **(A, B)**, the Wilcoxon test for continuous variables **(C)** and the log-rank test for time-to-event analysis **(D)**. CMV, human cytomegalovirus; HSCT, hematopoietic stem cell transplantation.

**Figure 6:**
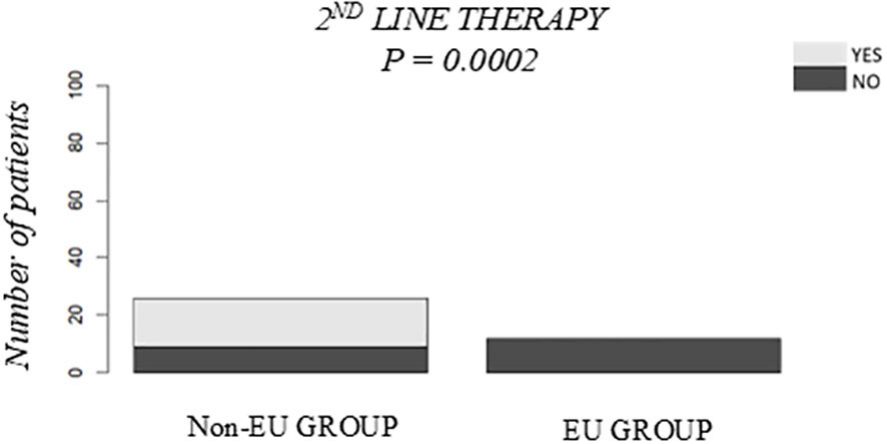
Comparison of second-line therapy required in the Non-European vs. the European Groups within the Study Group. P-values were obtained using the χ2 test.

First-blood CMV DNA detection after HSCT was lower in EU patients (28.1% vs. 64.1%, p = 0.006) (**Figure 5A**). No EU patient experienced second-blood CMV DNA detection, while 23.7% of non-European patients did (p = 0.0032) (**Figure 5B**). Median CMV DNA viral load were lower in EU patients (772 vs. 4710 copies/mL, p = 4e-04) (**Figure 5C**), with faster viral clearance (p < 0.0001) (**Figure 5D**). Seventeen non-European patients required second-line therapy, while none in the EU group did (p = 0.0002) (**Figure 6**). Acute and chronic GVHD incidence did not differ (p = 0.1555 and p = 0.2929, respectively) (**Supplementary Figure 3**).

### 3.6 CMV disease incidence and outcomes

Due to the limited number of patients developing CMV disease (9/141), it was not possible to formally analyze differences in incidence and severity between groups. Five patients were in the Study Group (3 EU, 2 non-European), and four were in the Control Group. All Study Group patients received CMV-specific Ig as salvage therapy. Three patients in the EU subgroup were cured (two with CMV pneumonia and severe renal dysfunction, one with encephalitis) (22). Two non-European patients recovered only after undergoing advanced-line treatments.

### 3.7 Impact of CMV-specific Ig prophylaxis in non-European versus control groups

No significant differences were observed when comparing non-European (from Study Group) and historical Control Groups in terms of first CMV DNA positivity, reactivation, CMV DNA viral load, treatment duration, or second-line therapy. Hospital stay and acute or chronic GVHD incidence were also similar. Immunological recovery at day +90 was significantly higher in the non-European Group (p = 0.0039).

### 3.8 Population pharmacokinetics of CMV-specific Ig

To investigate factors associated with group differences, a POP/PK analysis was performed using data from 38 patients (25 males, 13 females; mean age 8.88 ± 5.82 years) within 90 days post-HSCT (23). The dataset included 188 CMV-specific Ig doses and 173 observations collected from 24 hours to 42 days post-dose. A 2-compartment model with additive error was fitted, incorporating height (HGT) and body weight (BW) on clearance (Cl) and peripheral volume (V2), respectively:

Cl (L/day) = 0.0175 x (HGT/139)^0.474^
V1 (L) = 0.0804
intercompartmental clearance (Q) (L/day) = 0.0121
V2 (L) = 9.43 x (BW/29.3)^0.44^

Mean Cl and t1/2 were 0.018 ± 0.006 L/day and 14.98 ± 5.19 days respectively, consistent with literature (23). Over repeated doses, Cl increased (from 0.015 ± 0.005 up to 0.018 ± 0.006 L/day, ANOVA F = 3.473, p < 0.01) and t1/2 decreased significantly (from 17.74 ± 5.72 up to 13.68 ± 3.73 days, ANOVA F = 3.855, p < 0.01). Notably, seven patients with higher Cl (0.025 ± 0.005 L/day) developed or were affected by acute GVHD. Inter- and intra-patient variability was included (**Table 2**; **Supplementary Figures 5**–**7**). No sex differences were observed.

**Table 2:** Population values of pharmacokinetic parameters obtained by the final POP/PK model.

Parameter	VALUE	S.E.	R.S.E. (%)
Cl (L/day)	0.018	0.003	19.6
V1 (L)	0.080	0.217	37.8
Q (L/day)	0.012	0.066	40.5
V2 (L)	9.430	0.066	28.6
IIVCl	0.271	0.047	17.5
IOVCl	0.266	0.056	21.1
Additive error (mg/L)	13.1	2.550	19.5

SE, standard error of the mean; RSE, relative standard error; Cl, V_1_, Q, V_2_, systemic clearance, central volume of distribution, intercompartmental clearance, peripheral volume of distribution, respectively; IIV, interindividual variability; IOV, interoccasion variability.

## 4 Discussion

Current guidelines for CMV management after HSCT do not recommend CMV-specific Ig for prophylaxis or pre-emptive therapy (21, 24–26). However, recent studies in both pediatric and adult populations have shown that these Ig significantly reduce CMV infection and disease incidence without the adverse effects commonly associated with standard antiviral therapies (27–30). Unlike antiviral drugs, which cannot prevent free virus particles from infecting healthy cells (31), CMV-specific polyclonal Ig, such as CMV-specific Ig, bind CMV surface antigens, inhibit viral entry, mediate complement-dependent lysis, promote opsonization and phagocytosis, induce antibody-dependent cellular cytotoxicity, and enhance immunomodulation by reducing cytokine secretion. *In vitro* and clinical studies have shown that CMV-specific Ig decreases the production of IL-2, IFN-γ, IL-6, and IL-10, and it may prevent CMV binding to target cells until the emergence of CD8+ anti-CMV lymphocytes, thereby effectively controlling infection (32–37). The present study supports that anti CMV-specific Ig prophylaxis in pediatric allo-HSCT recipients reduces the incidence and severity of second-blood CMV DNA detection post-HSCT and enhances immune recovery without drug-related adverse events. These findings are consistent with previous reports, although the effects observed in this study are somewhat more modest (28, 29, 38). It is worth noting that the literature contains evidence regarding CMV-specific Ig, including studies conducted in various clinical settings, such as among pregnant women and solid organ transplant recipients, where the results have been inconsistent or have not demonstrated a significant preventive benefit (39–41).

Supportive care measures, including infection management, were standardized according to established institutional guidelines for both study groups throughout the transplant course and follow-up period. This standardization was critical due to the retrospective nature of the comparison, as it ensured that differences in clinical outcomes between groups were attributable to the prophylactic CMV-specific Ig strategy rather than variations in supportive care.

Genetic variability in CMV genomes is higher than in any other human herpesvirus (7), complicating vaccine and antiviral development due to potential viral resistance. The high within-host recombination rates and frequent co-infection with multiple strains likely drive this diversity (42, 43). CMV genomes have been analyzed in transplant recipients and congenital CMV patients across Europe, Australia, and the USA (44). Commercial anti-CMV Ig are derived from human plasma collected from local volunteer donors, likely reflecting regional CMV genotypes. The present study compared outcomes of CMV-specific Ig prophylaxis between patients from Europe and those from other continents. Although hospital stays were similar, the non-European Group showed a higher rate of initial CMV DNA detection, a greater CMV DNA viral load, and a longer duration to clearance. European patients experienced no second-blood CMV detection after HSCT and did not require second-line antiviral therapy. Although genomic sequencing was not performed, recent studies suggest the presence of distinct CMV genotypes in Europe versus Africa, which supports our hypothesis that ethnic and viral diversity impact the efficacy of prophylaxis (45).

Optimal dosing for CMV-specific Ig remains undefined. The Italian Medicines Agency (AIFA) recommends 1 mL (100 U)/kg for all patients (46). However, deviations from these standards are frequently observed in real-world clinical practice (23, 28, 29, 38, 47, 48). The present study monitored anti-CMV IgG concentrations before and after CMV-specific Ig administration to confirm the effectiveness of the protocol. Notably, early post-HSCT recipients were unable to produce endogenous antibodies, possibly due to delayed B-cell reconstitution, as previously reported (49).

A population pharmacokinetic (POP/PK) analysis was conducted on a cohort of 38 patients, which enabled the development of a two-compartment model with satisfactory fit, estimating a terminal half-life (t1/2) of approximately 15 days with moderate variability (>30%). The obtained t1/2 was longer than that reported in other studies, possibly due to longer sampling intervals (up to 42 days), infections, or higher viral loads (24, 50, 51). However, no clear pharmacokinetic/pharmacodynamic relationship between CMV-specific Ig exposure and clinical or virological outcomes was observed, likely reflecting interindividual variability in both IgG clearance and viral replication. Notably, t1/2 decreased across the initial five dosing occasions (up to 92 days), consistent with immune reconstitution and evolving viral activity. The present data confirm that acute GVHD, particularly when accompanied by intestinal involvement, may contribute to increased IgG clearance via albumin loss, as previously observed by Guiot et al. (52).

This study is the first to evaluate the efficacy of European-derived anti-CMV IgG prophylaxis in pediatric HSCT recipients from diverse ethnic backgrounds. Our findings indicate that while significant clinical benefits—such as reduced CMV reactivation and enhanced immune recovery—are maintained, protection levels and viral clearance kinetics vary. Rather than a lack of effect, the data reveal a reduced efficacy profile, likely resulting from diminished recognition of region-specific CMV variable epitopes. These results suggest that geographic strain mismatching directly influences the overall magnitude of protection. However, several methodological limitations must be acknowledged. The use of a historical Control Group, though necessary, introduces an intrinsic constraint compared to a randomized controlled trial, particularly with regard to the potential for temporal heterogeneity in supportive care protocols. Nevertheless, the prospective cohort (Study Group) was rigorously selected and deemed suitable for evaluating the clinical impact of the prophylaxis in this homogeneous, high-risk pediatric population, as significant differences in infection endpoints could be reasonably attributed to the intervention. A further limitation is the absence of CMV genotyping, which precludes confirmation of the potential role of viral strain diversity in influencing the observed outcomes across different ethnic groups. Additionally, potential confounding factors weren’t statistically analyzed due to our small cohort size, limiting robust multivariate analysis. Moreover, POP/PK data may be affected by the modest sample size (38 children) and the limitations of the ELISA assays in distinguishing endogenous from exogenous anti-CMV antibodies. To minimize confounding, the analysis was restricted to observations within 90 days post-HSCT, a period in which antibody production is minimal (53).

To the best of our knowledge, this study is the first to evaluate prophylaxis with European-derived anti CMV-specific Ig in pediatric HSCT recipients of diverse ethnic backgrounds. Results show substantial benefits of the prophylaxis in reducing CMV reactivation and supporting immune recovery. The observed differences in prophylaxis efficacy between EU and non-European patients suggest that CMV genome variability and host factors may influence clinical outcomes. Future research should include larger, multi-ethnic cohorts, incorporate viral genotyping, and refine dosing strategies to optimize prophylaxis.

## 5 Conclusions

Due to the ongoing clinical challenges posed by CMV in pediatric HSCT and the uncertainties concerning the protective role and optimal application of anti-CMV-specific Ig, a comprehensive investigation is crucial. Our proposed approach combines detailed clinical outcome assessment with a first-of-its-kind POP/PK analysis. This unique dataset directly addresses a gap in current prophylactic strategies in this vulnerable population. By rigorously evaluating both the clinical efficacy and the factors governing drug exposure, we expect our findings to move beyond the current controversy and provide evidence-based guidelines for the rational and optimized dosing of anti CMV-specific Ig. Ultimately, the aim is to improve post-transplant outcomes in pediatric recipients globally.

Overall, CMV-specific Ig prophylaxis represents an effective adjunct to standard HSCT care, improving viral control and immune reconstitution while avoiding the toxicity of antiviral drugs. These findings support its consideration in pediatric HSCT protocols and underscore the need for further investigation into dosing, timing, and the impact of viral genetic diversity on prophylactic efficacy.

## Data Availability

The original contributions presented in the study are included in the article/**Supplementary Material**. Further inquiries can be directed to the corresponding author.
