# Supplementary material for: The diversity of cytomegalovirus among blood donors and transplant recipients could affect the effectiveness of specific anti-CMV immunoglobulins
Source: Front Immunol. 2026 Apr 29;17:1832716. doi: 10.3389/fimmu.2026.1832716 (PMC13167980; doi:10.3389/fimmu.2026.1832716)
Supplement: Supplementary file 1 [file Supplementaryfile1.docx]

Supplementary Material

# Supplementary Figures and Tables


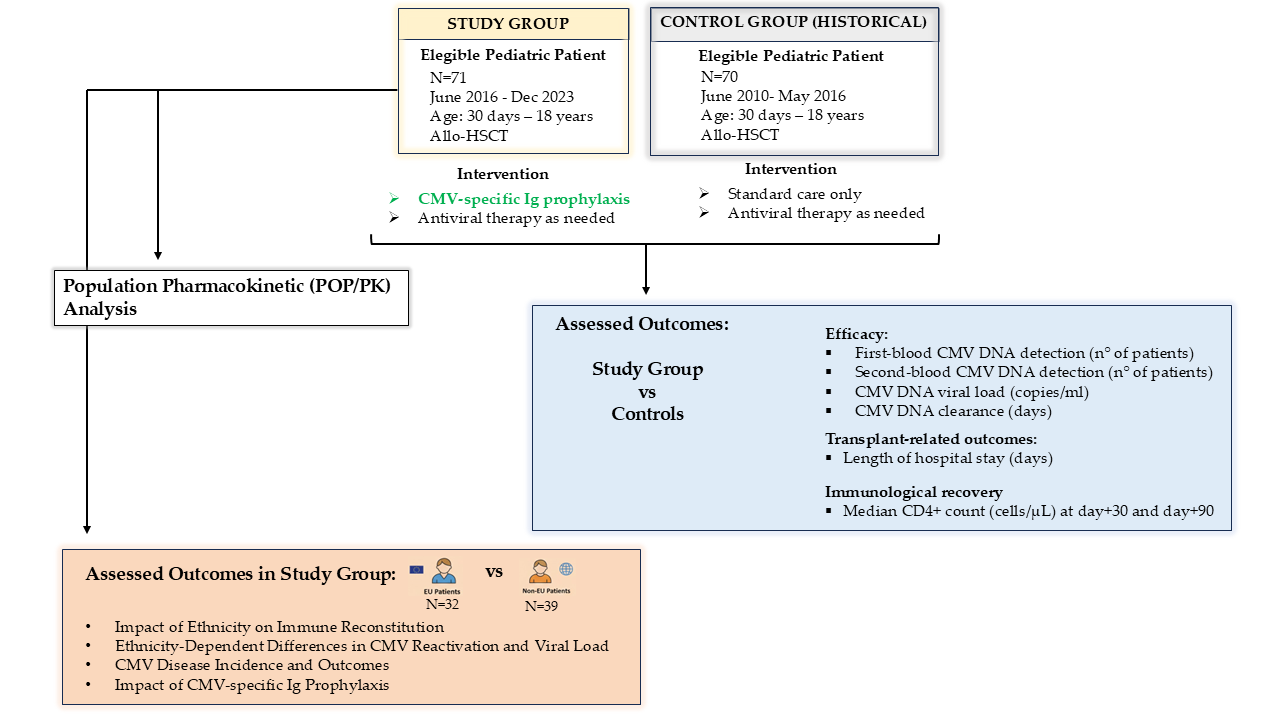


**Supplementary Fig.1. A schematic overview of the methods is presented, including: cohort classification, intervention protocol, and outcomes assessed.** CMV: human cytomegalovirus. Allo-HSCT: allogeneic hematopoietic stem cell transplantation.

**
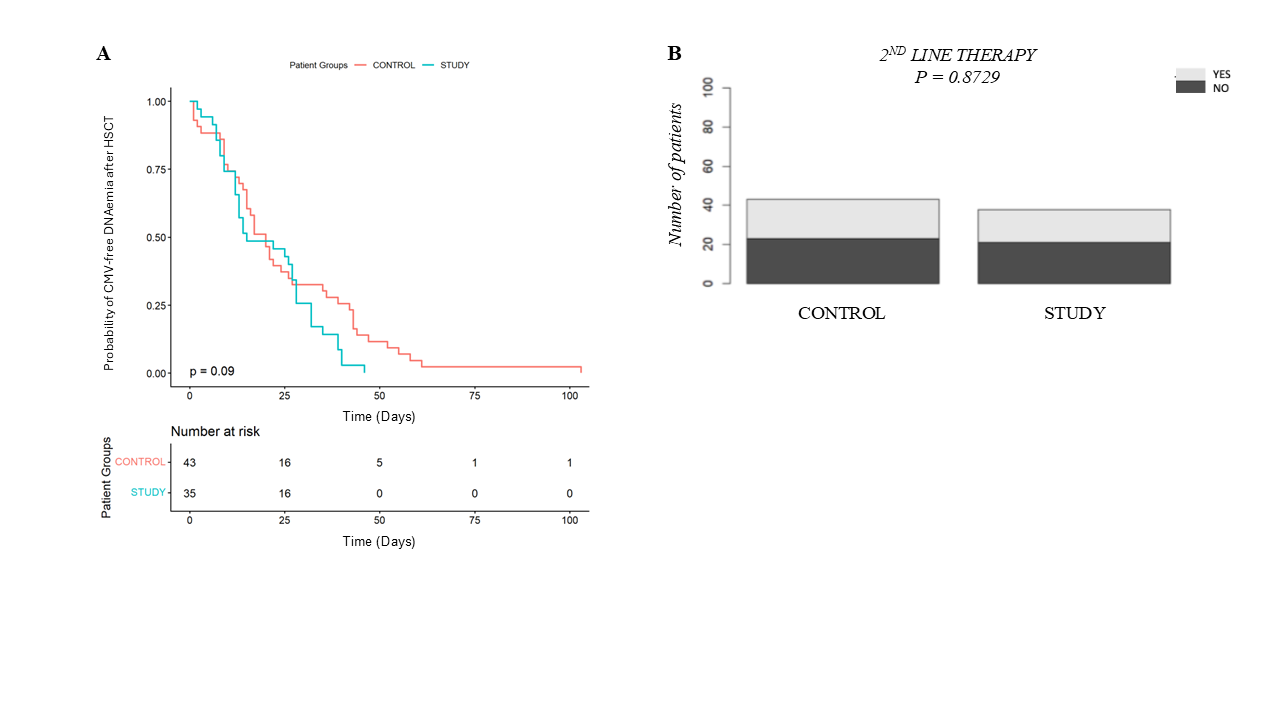
**

**Supplementary Fig.2. Kaplan-Meier estimates of the probability of CMV-free DNAemia after HSCT and second-line therapy in Control versus Study groups.** Kaplan–Meier curves compare the Control Group vs the Study Group regarding the time between CMV DNA detection during post-HSCT (Panel A) and the use of second-line therapy (Panel B). Control Group n=43, Study Group n=35. P-values were obtained using the log-rank test for time-to-event analysis (Panel A) and χ2 test for categorical variables (Panel B). CMV: human cytomegalovirus; HSCT: hematopoietic stem cell transplantation.


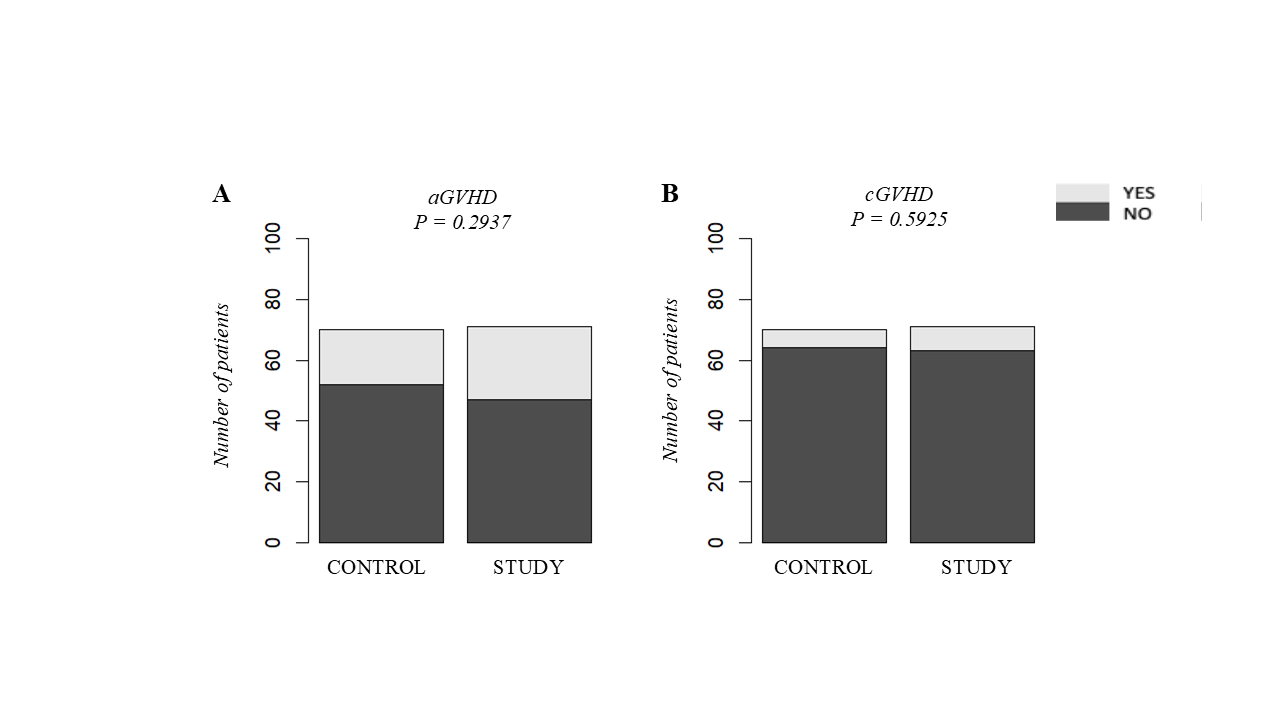


**Supplementary Fig.3. Comparison of acute GVHD (Panel A) and chronic GVHD (Panel B) incidence between the Control Group and the Study Group.** P-values were obtained using the χ^2^ test for categorical variables. aGVHD: acute graft-versus-host disease; cGVHD: cronic graft-versus-host disease.


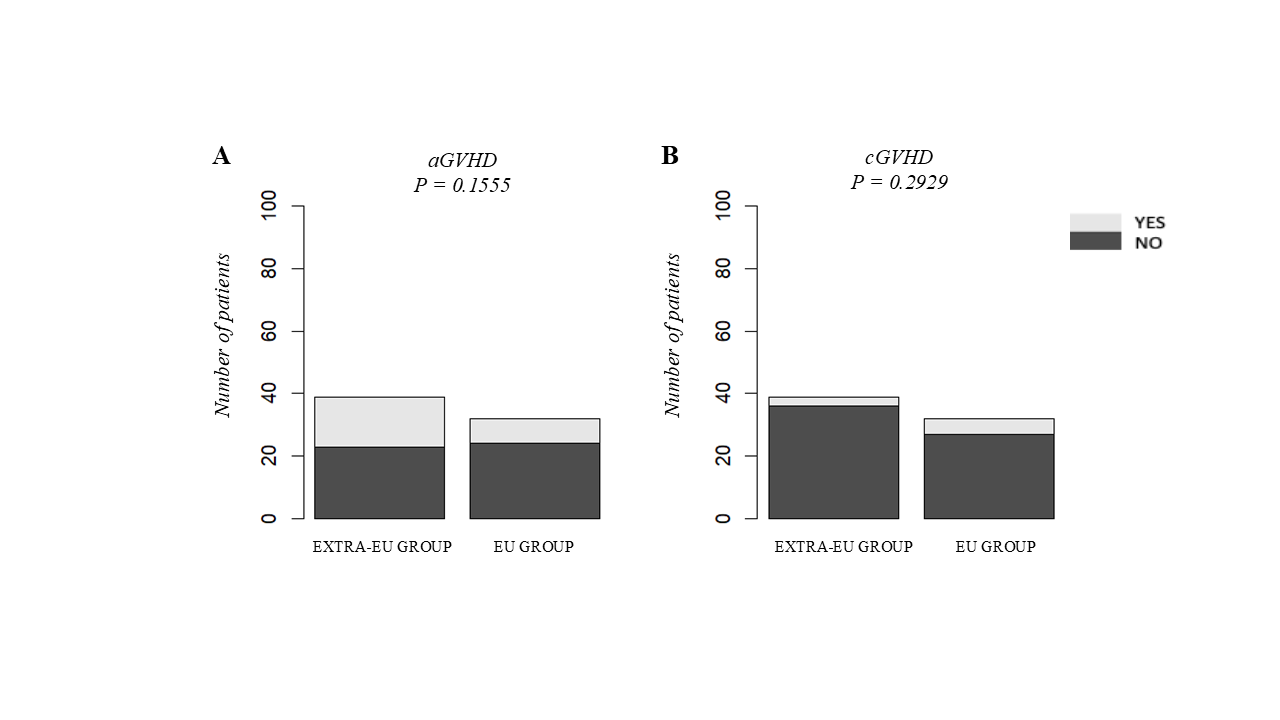


**Supplementary Fig.4. Comparison of acute GVHD (Panel A) and chronic GVHD (Panel B) incidence between the Extra-European and the European Groups.** P-values were obtained using the χ^2^ test for categorical variables. aGVHD: acute graft-versus-host disease; cGVHD: cronic graft-versus-host disease.


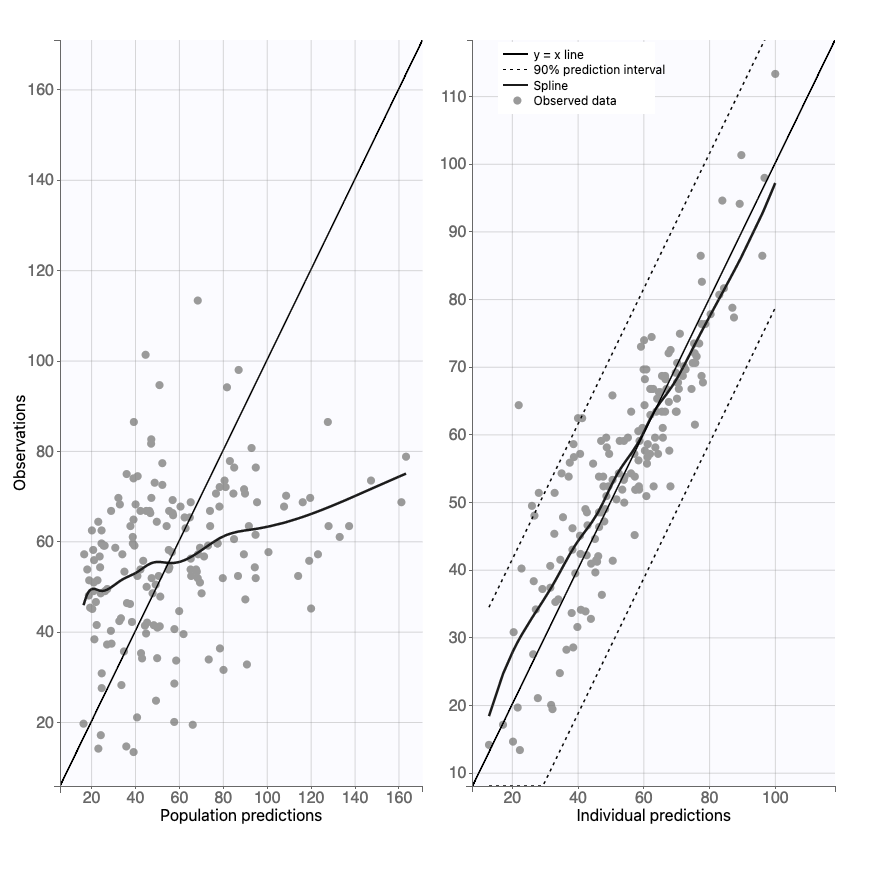


**Supplementary Fig.5. Goodness-of-plot graphs: correlation between observed values of serum concentrations of anti-CMV Ig, population (left), and individual (right) prediction.**


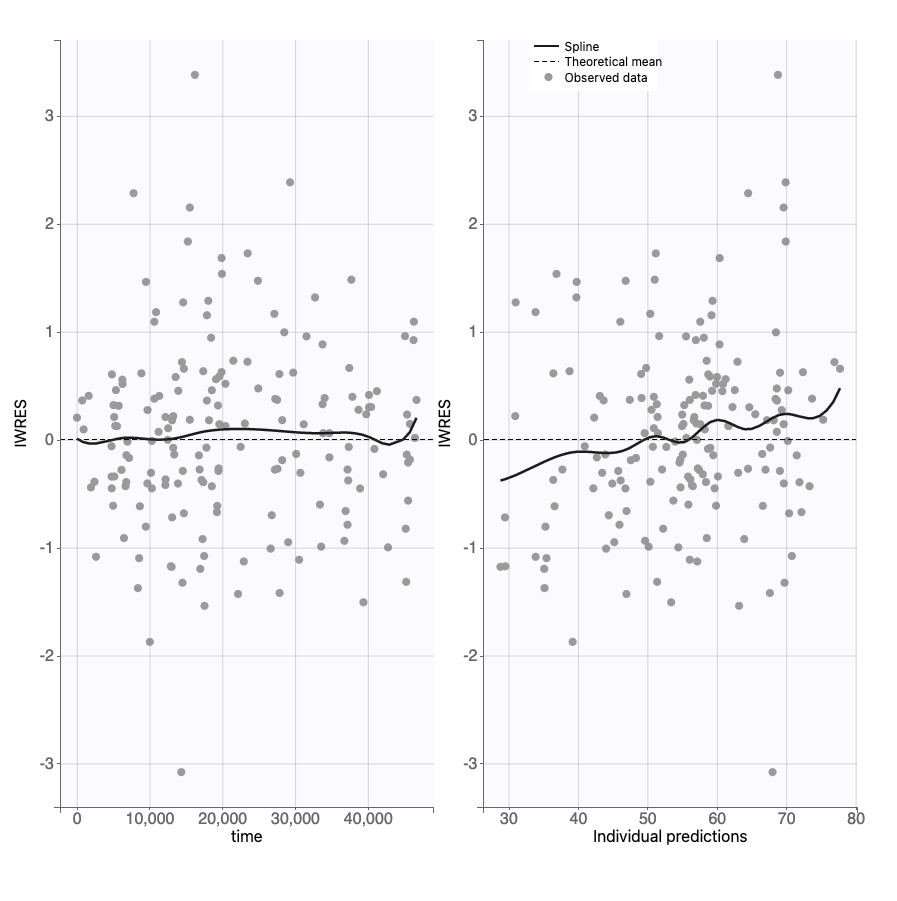


**Supplementary Fig.6.** **Goodness-of-plot graphs: Correlation between individual weighted residual (iWRES) values and time (left, h) and individual predictions (right).** Time (h) in the left plot refers to the time interval between the first dose of the first patient and the last measurement of the past patient (approximately 5.13 years).


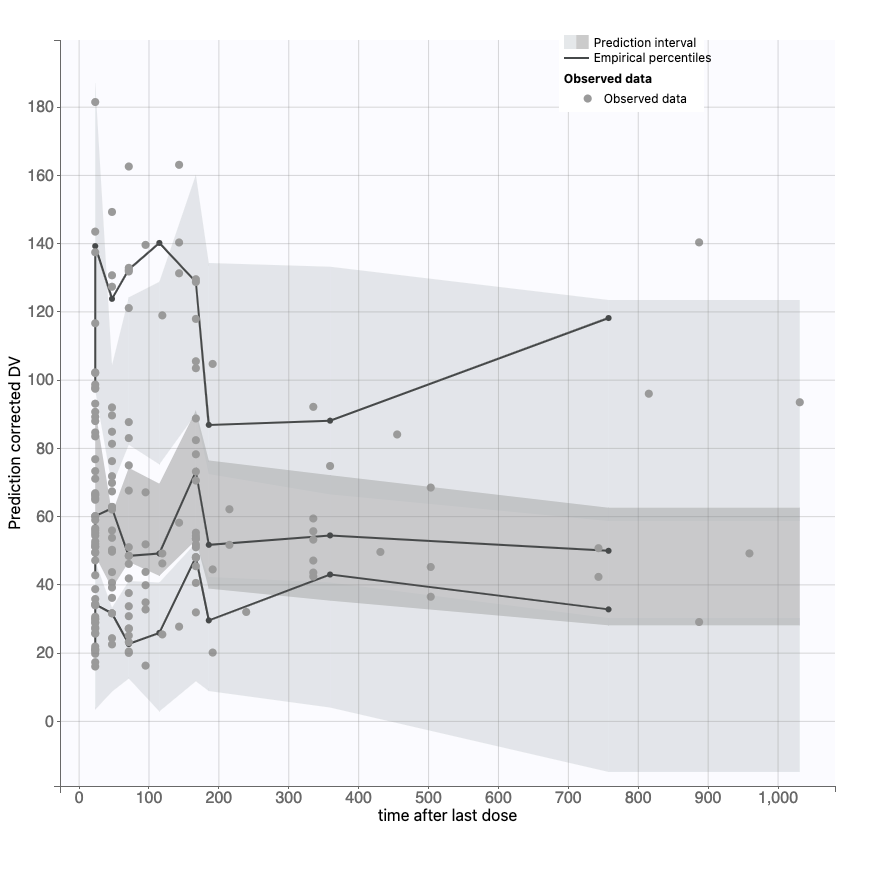


**Supplementary Fig.7.** **Prediction corrected visual predictive check (pcVPC) of prediction corrected measured plasma concentrations of anti-CMV Ig versus time (h).** The data refer to the first five occasions, which span the first three months after HSCT.
